# Using Unnatural Protein Fusions to Engineer a Coenzyme Self-Sufficiency System for D-Phenyllactic Acid Biosynthesis in *Escherichia coli*

**DOI:** 10.3389/fbioe.2021.795885

**Published:** 2021-12-17

**Authors:** Zhao Qin, Dan Wang, Ruoshi Luo, Tinglan Li, Xiaochao Xiong, Peng Chen

**Affiliations:** ^1^ School of Chemistry and Chemical Engineering, Chongqing University, Chongqing, China; ^2^ Department of Biological Systems Engineering, Washington State University, Pullman, WA, United States

**Keywords:** coenzyme self-sufficiency, d-Lactate dehydrogenase, phenyllactic acid, D-PLA, glycerol dehydrogenase, fusion protein

## Abstract

The biosynthetic production of D-penyllactic acid (D-PLA) is often affected by insufficient supply and regeneration of cofactors, leading to high production cost, and difficulty in industrialization. In this study, a D-lactate dehydrogenase (D-LDH) and glycerol dehydrogenase (GlyDH) co-expression system was constructed to achieve coenzyme NADH self-sufficiency and sustainable production of D-PLA. Using glycerol and sodium phenylpyruvate (PPA) as co-substrate, the *E. coli* BL21 (DE3) harboring a plasmid to co-express LfD-LDH and BmGlyDH produced 3.95 g/L D-PLA with a yield of 0.78 g/g PPA, similar to previous studies. Then, flexible linkers were used to construct fusion proteins composing of D-LDH and GlyDH. Under the optimal conditions, 5.87 g/L D-PLA was produced by expressing LfD-LDH-l_3_-BmGlyDH with a yield of 0.97 g/g PPA, which was 59.3% increased compared to expression of LfD-LDH. In a scaled-up reaction, a productivity of 5.83 g/L/h was reached. In this study, improving the bio-catalytic efficiency by artificial redox self-equilibrium system with a bifunctional fusion protein could reduce the bio-production cost of D-PLA, making this bio-production of D-PLA a more promising industrial technology.

## Introduction

Phenyllactic acid (PLA) widely exists in honey and fermented food and includes two enantiomers as D-phenyllactic acid (D-PLA) and L-phenyllactic acid (L-PLA) (Sorrentino et al., 2018; Luo et al., 2020a). Due to its safety, antimicrobial activity, low odor and good hydrophilicity, PLA has potential applications in the food, animal feed, pharmaceutical, and cosmetic industries (Valerio et al., 2016; Sun et al., 2019). Previous researchers found that the bactericidal effect of D-PLA was stronger than that of L-PLA (Dieuleveux et al., 1998) and D-PLA can be used as biological preservatives, antiviral compounds, hypoglycemic drugs, and protein inhibitors (Xu et al., 2015; Zhu et al., 2017; Liu et al., 2021). Moreover, because PLA can be polymerized into the aromatic polymers poly-PLA, it is a promising bio-based material (Wu et al., 2020). Although chemical methods of PLA production have been well studied, some limitations such as complex technology routes, harsh reaction conditions, excessive by-products and environmental pollution have hampered the chemical synthesis of PLA. (Wang et al., 2018a; Costa et al., 2020; Xu et al., 2020). Compared with chemical methods, biological methods have advantages of mild action conditions, energy savings, and environmental compatibility (Zhao et al., 2018). Therefore, the development of a eco-friendly biosynthesis method for PLA production is highly desirable.

PLA could be generally produced by a wide range of lactic acid bacteria or *Escherichia coli* with PPA, phenylalanine or glucose as starting materials in previous studies (Sorrentino et al., 2018; Wu et al., 2020). *L. plantarum* IMAU10124, *P. pentosaceus*, *E. coli* GK1, and *L. crustorum* NWAFU1078 produced 0.229 g/L (Zhang et al., 2014), 0.136 g/L (Yu et al., 2015a), 1.429 g/L (Kawaguchi et al., 2015), and 2.526 g/L (Xu et al., 2020) of PLA grown in MRS broth. Overall, since these methods produce various by-products and have relatively low yields, enzymatic/whole-cell cascade onecatalyst emerged as a green alternative for PLA production (Hou et al., 2019). Whole-cell catalytic conversion of PPA to PLA by strain overexpressing of NADH-dependent lactate dehydrogenase (LDH) is regarded as a more cost-efficient technology (Li et al., 2008; Mu et al., 2012; Luo et al., 2020b). D-LDH was overexpressed to produce D-PLA from PPA in *Leuconostoc mesenteroides* ATCC 8293, and the results showed that growing cells produced 35 mM D-PLA with a yield of 75.2–83.3% (Li et al., 2014). A novel NADH-dependent LDH gene, named *lrldh*, was cloned from *Lactobacillus rossiae* and heterologously expressed in recombinant *E. coli*/pET28a*-lrldh.* 20.5 g/L D-PLA was produced with a productivity of 49.2 g/L/d in a fed-batch biotransformation process (Luo et al., 2020a). Glucose and Phe need to undergo intermediate metabolism to produce PLA, which results in the increased fermentation time and decreased productivity. PPA is considered as a feasible precursor for the large-scale production of PLA, because it can biosynthesize PLA in one-step method and improve the catalytic efficiency (Li et al., 2014; Wang et al., 2018b).

However, the conversion from PPA to PLA by LDH requires the consumption of expensive coenzyme NADH. So the pathway involved in NADH regeneration is considered as an auxiliary pathway for PLA biosynthesis (Wu et al., 2020). Therefore, the whole-cell cascade catalysis using recombinant *E. coli* co-expression of glucose dehydrogenase (GDH)/formate dehydrogenase (FDH) and LDH has been widely used in the synthesis of PLA (Zhao et al., 2018; Rajanikar et al., 2021). Recombinant *E. coli* co-expressing LDH and GDH produced 17.25 g/L PLA from PPA with a productivity of 0.86 g/L/h (Zhu et al., 2017). Recombinant *E. coli* co-expressing D-LDH and FDH produced 10.02 g/L D-PLA from phenylalanine with a productivity of 1.67 g/L/h (Zheng et al., 2018). Unlike FDH or GDH, glycerol dehydrogenase (GlyDH) catalyzes the reduction of glycerol to dihydroxyacetone without the formation of toxic formic acid or gluconic acid (Xu et al., 2016), therefore, among these whole-cell reaction systems, co-expression of GlyDH and LDH might be a novel strategy for PLA synthesis. Furthermore, the co-substrate glucose was replaced with cost-effective glycerol to decrease the cost and improve the intracellular cofactor concentration.

Fusion proteins have been emerged recently as a new technology in biocatalysts, protein switches and therapeutics, aiming to channel substrates in sequential reactions, reduce effective reaction volume, facilitate cofactor regeneration and improve electron transfer (Yu et al., 2015b; Aalbers and Fraaije, 2019; Wu et al., 2021). Linkers have a critical role in the functionality and bioactivity of the destined fusion proteins, which can increase the stability/folding, expression, improve the biological activity, target to specific sites (Anami et al., 2018; He et al., 2019). Fan *et al.* constructed bifunctional and trifunctional fusions Mdh-Hps, Hps-Phi, and Mdh-Hps-Phi with flexible linkers (GGGGS)_3_ and (GGGGS)_6_, and the results showed that fusing Mdh with Hps or Hps-Phi enhanced methanol conversion to fructose-6-phosphate by 30% (Fan et al., 2018). Patgiri *et al.* engineered a fusion protein composed of lactate oxidase and catalase, which normalized the intracellular NADH:NAD + ratio by converting lactate and oxygen to pyruvate and water (Patgiri et al., 2020).

**FIGURE 1:**
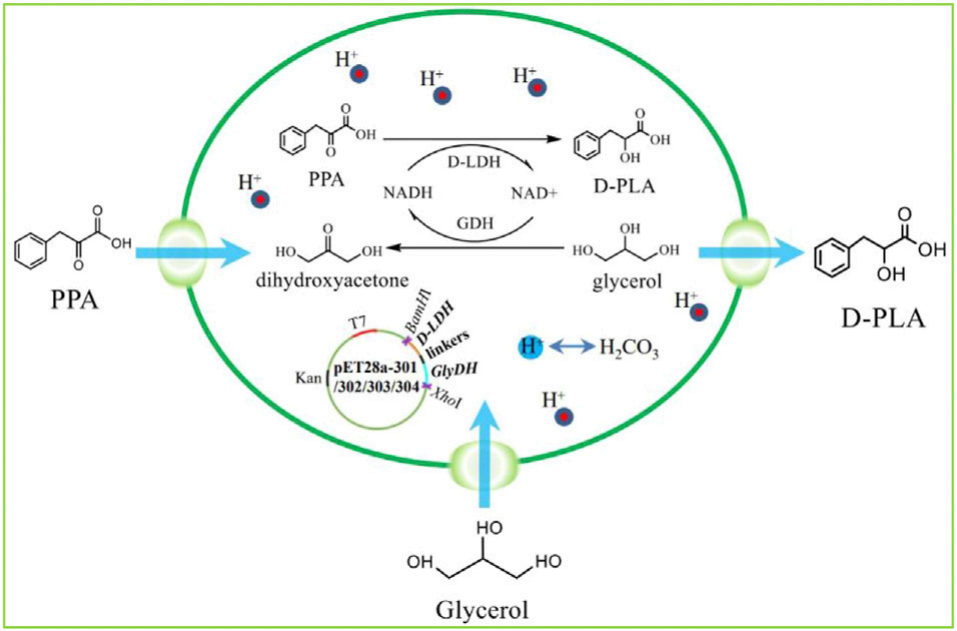
The program of redox self-balanced coenzyme regeneration and whole-cell synthesis of D-PLA.

In this study, fusion protein engineering of D-LDH and GlyDH is conducted to aim for effective regeneration of coenzyme NADH for D-PLA biosynthesis. Firstly, a NADH-dependent D-LDH was overexpressed in recombinant *E. coli* CP101 to produce D-PLA from PPA. Then, plasmids for co-expression of *D-LDH* (two different genes) and *GlyDH* (two different genes) were transformed in *E. coli* (CP201, CP202, CP203, CP204) to achieve coenzyme self-sufficiency. To further improve the regeneration efficiency of coenzyme and to increase the yield of D-PLA, artificial fusion protein technology was used to construct D-LDH and GlyDH bifunctional fusion proteins as shown in Figure 1.

## Materials and Methods

### Strains, Plasmids, and Chemicals

Enzymes (DNA polymerases, T4 DNA ligase, *BamH*I, and *Xho*I) and kits (DNA purification kit, plasmid isolation, DNA ligation kit, and competent cell preparation kit) were supplied by Takara (Dalian, China). With the exception of PPA, PLA, and NADH (Tsingke Biotechnology Co. Ltd., Beiing, China), all chemical reagents were purchased from China National Medicines Co. Ltd. (Beijing, China).

### The Construction Process of the Plasmids

All bacterial strains and plasmids used in this study are listed in Supplementary Table S1 *.* The *D-LDH* genes *of Lactobacillus fermentum*, *Lactobacillus sp*.SK007, and *GlyDH* genes of *Bacillus megaterium*, *Exiguobacterium sibiricum* were amplified via polymerase chain reaction (PCR) by using the relevant primer pairs listed in Supplementary Table S3. The PCR products were digested with *BamH*I and *Xho*I and ligated to pET28a to construct pET28a-*LfD-LDH*, pET28a-*LsD-LDH*, pET28a-*BmGlyDH* and pET28a-*EsGlyDH*, and then introduced into BL21 (DE3) individually to form the novel corresponding recombinant strains CP101, CP102, CP103, and CP104. The *LfD-LDH* and Bm*GlyDH* were ligated by fusion PCR, and then the PCR product was digested with *BamHI* and *XhoI* and ligated to pET28a to construct pET28a-*LfD-LDH-BmGlyDH.* Using the same method as above, plasmids pET28a-*LfD-LDH-EsGlyDH*, pET28a-*LsD-LDH-BmGlyDH*, and pET28a-*LsD-LDH-EsGlyDH* could be constructed. Subsequently, plasmids above were introduced into BL21 (DE3) individually to form the novel corresponding recombinant strains CP201, CP202, CP203, and CP204. To construct fused *D-LDH-GlyDH* genes, fragments encoding flexible linkers were added to the C-terminal of *D-LDH* and N-terminal of *GlyDH* by overlap extension PCR using the primer pairs listed in Supplementary Table S3. Flexible linkers (GGGGS)_1_ (GGGGS)_2_, (GGGGS)_3_, and (GGGGS)_6_ listed in Supplementary Table S2 were added to the C-terminal of *LfD-LDH* and N-terminal of *BmGlyDH* and then ligated to pET28a to construct pET28a-*LfD-LDH-l*_*1*_ *-BmGlyDH*, pET28a-*LfD-LDH-l*_*2*_ *-BmGlyDH*, pET28a-*LfD-LDH-l*_*3*_ *-BmGlyDH*, and pET28a-*LfD-LDH-l*_*6*_ *-BmGlyDH*, and then introduced into BL21 (DE3) individually to construct recombinant strains CP301, CP302, CP303, and CP304. Successfully constructed recombinant plasmids were verified by DNA sequencing (Tsingke Biotechnology Co., Ltd., Beijing, China). CP100 carrying the backbone plasmid pET28a was constructed as the control strain.

### Preparation of Whole-Cell Biocatalyst

Recombinant *E. coli* were inoculated into 40 ml LB medium (10 g/L tryptone, 5 g/L yeast extract, 10 g/L NaCl) containing kanamycin (50 μg/ml) and grown in a rotary shaker (200 RPM) at 37°C overnight. 400 µL seed culture was inoculated into 40 ml LB medium containing 50 µg/ml kanamycin, and incubated at 37°C and 200 RPM to OD_600_ = 0.8. The recombinant *E. coli* was induced with 0.2 mM isopropyl-β-D-thiogalactoside (IPTG) at 25°C for 10 h. The cells were obtained via centrifugation at 4°C and 6,000 r/min for 10 min and washed three times with pH 7.0 PBS buffer. Cell catalyst (DCW) concentration was checked spectrophotometrically (722s, Shanghai Precision Scientific Instrument Co., Ltd., Shanghai, China) at an optical density of 600 (Yang et al., 2013). The concentrations of NADH, PPA, and PLA were measured as reported previously (Hou et al., 2017).

### Optimization of D-LDH Induction and Enzymatic Catalysis Conditions

The recombinant *E. coli* was inoculated into 40 ml LB medium containing kanamycin (50 μg/ml) and grown overnight in a rotary shaker (200 RPM) at 37°C. 400 µL seed culture was inoculated into 50 ml LB medium for expression. The induction conditions, including temperature (20°C, 25°C, 30°C, 35°C, and 40°C), pH (6.0, 6.5, 7.0, 7.5, and 8.0), and concentration of IPTG (0.1, 0.2, 0.3, 0.4 and 0.5 mM), were investigated.

The enzymatic catalysis conditions of PPA to D-PLA were optimized. For D-PLA bioconversion, the catalytic system had 5 ml, which included 5 g/L PPA, 5.61 g/L glycerol, 9 g/L DCW, and 2% glucose. The bioconversion reactions were performed at 35°C on a 200 RPM shaker for 10 min for D-PLA production. The effects of temperature (25°C, 30°C, 35°C, 37°C, and 40°C), pH (6.0, 6.5, 7.0, 7.5, and 8.0), and PPA concentration (5, 6, 7, 8, and 9 g/L) on D-PLA production were determined.

### Scale-Up of Bioconversions

Scale-up biotransformation is usually achieved by fed-batch cultures in a bioreactor (Abadli et al., 2021; Parizotto et al., 2021). The cell culture was concentrated and suspended (OD_600_ = 1.6) in 1-L PBS buffer (pH 7.0) with 60 g/L PPA and 66.5 g/L glycerol. Additional 40 g/L PPA and 16.78 g/L glycerol were added after 8 h bioconversion. The total concentration of PPA used in the system was 100 g/L. The scaled-up bioconversion was performed in a 5-L fermenter at 200 RPM, pH 7.0, 35°C.

### Analytical Methods

The successful synthesis of PLA was confirmed by HPLC-MS (Agilent1260 series, Hewlett-Packard) with a C-18 5 µm column (4.6*25 mm) and the analysis was performed at 40°C with a mobile phase comprising 20% acetonitrile in water at a flow rate of 1 ml/min, wavelength 210 nm, 10 µL injection volume (Cheng et al., 2020) (Supplementary Figures S1, S2).

## Results and Discussion

### Construction of the D-PLA Biosynthetic Pathway in *E. coli*

**FIGURE 2:**
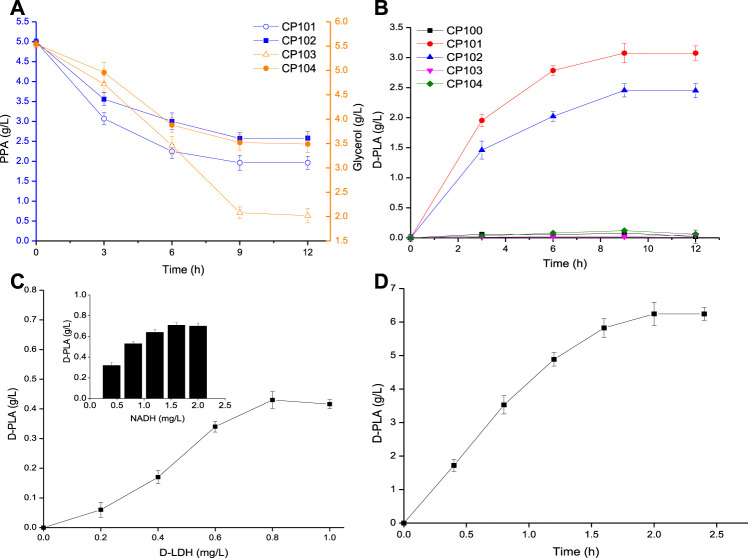
**(A)** Time course of the consumption of PPA (by CP101, CP102), and glycerol (by CP103, CP104); **(B)** Time course of the production of PLA by CP100, CP101, CP102, CP103, and CP104; **(C)** The effect of D-LDH and NADH concentration on D-PLA titer; **(D)** Time course of the production of D-PLA by enzyme catalysis. Data are means ± SD (*n* = 3).

Firstly, the synthesis pathway of D-PLA was constructed in single-enzyme expressing strains by overexpressing the D-LDH or GlyDH, so as to select suitable genes for the construction of co-expressing strains as shown in Figure 2A. CP101 with *Lf*D-LDH and CP103 with *Bm*GlyDH showed better productivity than CP102 with *Ls*D-LDH and CP104 with *Es*GlyDH, with maximum conversion rates of 60.7 and 70.4% for PPA and glycerol, respectively, within 12 h. At the same time, their production capacity to the D-PLA was measured as shown in Figure 2B. CP101 could achieve the highest yield of D-PLA (5 g/L PPA to 3.07 g/L D-PLA) without co-expression of a cofactor regeneration system, while CP100, CP103 and CP104 lacking *D-LDH* gene could hardly produce PLA as reported (Jung et al., 2019). No D-PLA was detected in the control system, indicating that no enzyme could catalyze PPA to D-PLA in CP100. Therefore, one-step biosynthesis of D-PLA using PPA as raw material has been successfully realized.

D-LDH is a NADH-dependent enzyme, which has commonly used in the biosynthesis of D-PLA (Li et al., 2008; Mu et al., 2012). The effects of D-LDH and NADH concentration on D-PLA titer were determined. As it could be seen from Figure 2C, with the increase of enzyme dosage, D-PLA increased up to a maximum of 0.43 g/L at 0.8 mg/L D-LDH and 0.71 g/L at 1.6 mg/L NADH within 10 min, respectively. D-PLA cannot be produced without NADH addition indicating that NADH-dependent D-LDH must rely on NADH to catalyze the conversion of PPA to D-PLA. Similar studies have reported that the enzymatic production of PLA by *P. pentosaceus* improved by 30-folds on supplementation with NADH and NADH-regeneration catalyst (Yu et al., 2014). As it could be seen from Figure 2D, under the optimal conditions at pH 7 and 35°C, with PPA 7 g/L, 0.8 mg/L D-LDH, and NADH 1.6 mg/L in the fermentation broth, the D-PLA titer reached 6.24 g/L with a yield of 0.881 g/g PPA, respectively, within 2 h. Although it has been proved that the production of D-PLA by D-LDH enzyme catalysis can be achieved by adding coenzyme NADH, the high cost of NADH limits its industrial application.

### The Self-Sufficient System of NADH With Coordinated Enzyme

**FIGURE 3:**
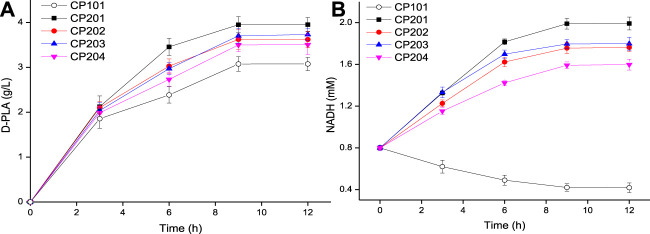
**(A)** Time course of the production of D-PLA by D-LDH and GlyDH co-expressed strains CP201, CP202, CP203, and CP204; **(B)** Time course of intracellular NADH concentration. Data are means ± SD (*n* = 3).

In order to make up for the shortage of NADH in single-enzyme expressing strains and further increase yield of D-PLA in recombinant *E. coli*, a co-expression system was constructed for PLA biosynthesis. As it could be seen from Figure 3A, the co-expressing strains CP201, CP202, CP203, and CP204 catalyzed the synthesis of D-PLA, and obtained 3.95, 3.62, 3.73, and 3.50 g/L D-PLA from 5 g/L PPA in the biotransformation process for 12 h, respectively, while the control CP101 only obtained 3.07g/L. Therefore, for the single-enzyme expression of D-LDH, the co-expression of D-LDH and GlyDH can increase the yield of D-PLA by about 29.7%.

The above enzymatic catalysis results (Figure 2C) show that the concentration of NADH is an important limiting factor affecting the production of PLA by D-LDH catalysis. The cost of using NADH was expensive, so the intracellular NADH concentration was increased by using co-substrates (Hou et al., 2019). Therefore, the NADH concentration can be increased by using co-substrates (PPA and glycerol) in the co-expression system. The concentration of NADH in whole-cell *E. coli* was measured during the biosynthesis of D-PLA as shown in Figure 3B. In the control strain CP101 with PPA as the substrate of D-LDH single-enzyme expression, NADH decreased by 47.8% at 9 h. However, the intracellular NADH concentration of co-expressing strains (CP201, CP202, CP203, and CP204) with PPA and glycerol as co-substrates showed an increasing trend. This is because GlyDH in co-expressing strains oxidizes glycerol to dihydroxyacetone and produces coenzyme NADH, so more abundant NADH can be provided. A similar study reported whole-cell biocatalyst of co-expressing GDH and D-LDH was constructed to produce 262.8 g/L/d D-PLA without supplement of NADH (Luo et al., 2020b). Therefore, the co-substrate and co-expression system successfully constructed in this study can realize the regeneration of NADH to better produce D-PLA.

### Improve the Efficiency of the Self-Sufficient System by Fusion Enzyme Engineering

In the area of enzyme engineering, early attempts were made to create two-protein fusions either to increase consecutive enzyme reaction rates or to generate bifunctional enzymes (Lindbladh et al., 1992; Beguin, 1999; Xiong et al., 2021). For example, a 77-fold improvement in the final product titer using a synthetic scaffold protein by recruiting three heterologous pathway enzymes in a designable manner (Dueber et al., 2009). For another example, a bifunctional enzyme coupling dihydroxyacetone kinase and fructose-1,6-bisphosphate aldolase was constructed to promote a 20-fold increase in the initial rate of the overall aldol reaction (Iturrate et al., 2010). According to previous studies, fusion enzyme engineering is a promising method for *in situ* recovery of co-enzyme NADH (Prachayasittikul et al., 2006).

**FIGURE 4:**
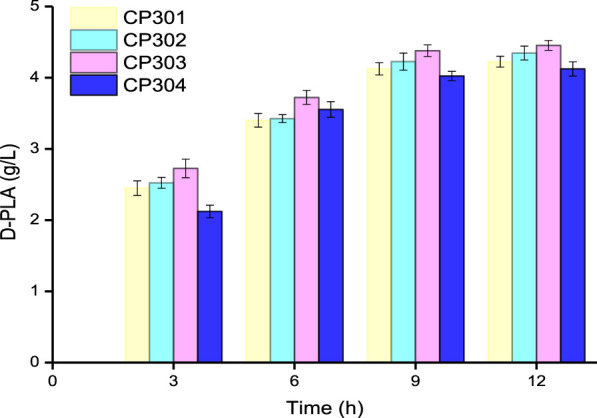
Time course of the production of D-PLA by fusion protein strains CP301, CP302, CP303, and CP304. Data are means ± SD (*n* = 3).

Based on the results of the above researchers in the area of fusion enzyme engineering, flexible Linkers (GGGGS)_1_, (GGGGS)_2_, (GGGGS)_3_, and (GGGGS)_6_ were used to construct D-LDH and GlyDH bifunctional fusion proteins by over-lap PCR technique. As it could be seen from Figure 4, the four fusion protein strains CP301 (*LfD-LDH-l*_*1*_ *-BmGlyDH*), CP302 (*LfD-LDH-l*_*2*_ *-BmGlyDH*), CP303 (*LfD-LDH-l*_*3*_ *-BmGlyDH*), and CP304 (*LfD-LDH-l*_*6*_ *-BmGlyDH*) were used to catalyze the transformation of 5 g/L PPA into D-PLA, and 4.23, 4.35, 4.45, and 4.12 g/L PLA were obtained, respectively, within 12 h. The strain CP303 achieved 4.45 g/L titer D-PLA with a yield of 0.879 g/g PPA, which is 1.13 times that of the strain CP201. This benefits from the flexible peptide linker which was likely to bring enzyme moieties in close proximity for superior cofactor channeling, making D-LDH and GlyDH catalysis more efficient (Dueber et al., 2009). A similar study reported the application of peptide linker in the construction of bifunctional FDH and leucine dehydrogenase enzymatic complex for efficient cofactor regeneration, showing the production rate of fusion enzymatic complex with suitable flexible peptide linker was increased by 1.2 times compared with free enzyme mixture (Zhang et al., 2017). In addition, this linker might provide the proper space and flexibility to accommodate the different subunits of the LDH and GlyDH, as dehydrogenases are typically multimeric, and the fusion of dehydrogenases has been found to perturb proper oligomerization (Lerchner et al., 2016; Aalbers and Fraajie, 2017; Peters et al., 2017). Therefore, we successfully constructed D-LDH and GlyDH bifunctional fusion proteins to accelerate the cyclic regeneration of NADH and improve the efficiency of enzyme catalysis.

### Optimization of Induction Conditions and Co-Substrates

In order to improve the ability and efficiency of the fusion proteins to catalyze the synthesis of D-PLA, the induction conditions of *E*. *coli* CP303 expressing D-LDH/GlyDH were optimized. Single factor experiments were used to optimize the induction conditions of recombinant *E. coli* in shake flasks, namely the influence of IPTG concentration, induction temperature, and pH on the catalytic synthesis of D-PLA by recombinant *E. coli* CP303. In addition, the ratio of substrate PPA, glycerol and cell catalyst were also optimized. Recombinant *E. coli* basic induction and co-substrate conditions: OD_600_ = 0.8, 0.1 mM IPTG, 5 g/L PPA, 5.61 g/L glycerol and induce expression at 25°C for 12 h.

**FIGURE 5:**
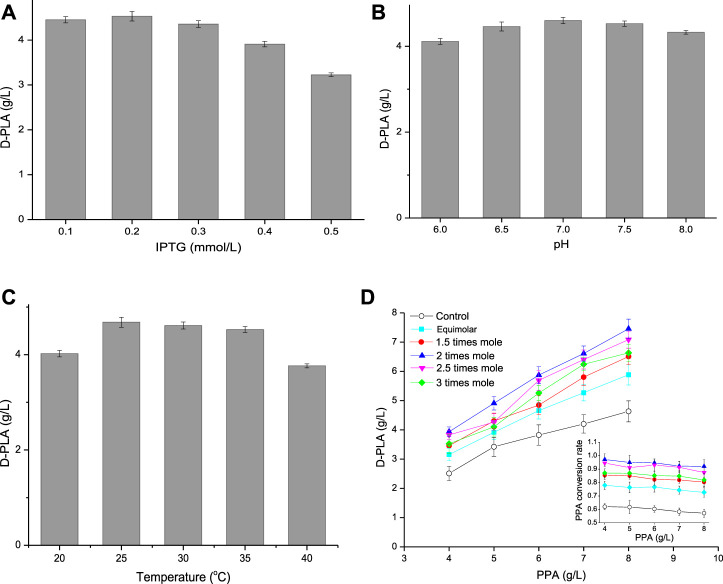
**(A)** Effect of IPTG concentration on D-PLA titer; **(B)** Effect of pH on D-PLA titer; **(C)** Effect of temperature on D-PLA titer; **(D)** Effect of substrate (glycerol, relative to PPA) on D-PLA titer. Data are means ± SD (*n* = 3).

As it could be seen from Figure 5A, when the IPTG concentration increased from 0.1 to 0.5 mM, the D-PLA titer increased first and then decreased. Compared with 0.1 mM IPTG, the expression of D-LDH/GlyDH fusion increased by induction with 0.2 mM IPTG and achieved a highest titer (4.53 g/L) D-PLA. However, when the IPTG concentration increased to 0.3 mM, it began to inhibit the growth of *E. coli* CP303, especially when the IPTG concentration was 0.5 mM. Therefore, the optimal IPTG concentration was 0.2 mM, the cell growth was better and D-LDH/GlyDH enzyme activity was the highest at this concentration. pH can affect the spatial structure and activity of enzymes, so an optimal pH needs to be explored. The effect of pH on D-PLA production as shown in Figure 5B. When the reaction system pH was 7.0, the D-PLA titer catalyzed by *E. coli* CP303 was higher than other pH, indicating that the neutral environment can maintain the good catalytic activity of D-LDH and GlyDH at the same time, so the high yield of D-PLA can be achieved by maintaining the pH at 7.0. Temperature affects the growth rate, cell metabolic activity and protein production rate of *E. coli* CP303, so induction temperature has also been studied as shown in Figure 5C. When the induction temperature was 20°C, the slow metabolism of recombinant *E. coli* led to the slow expression rate of D-LDH/GlyDH fusion protein and the low yield of D-PLA. When the induction temperature was higher than 30°C, the yield of D-PLA began to decrease, mainly because the fusion proteins at high temperature began to exist in the inclusion form. Therefore, the optimal induction temperature is 25°C, the highest titer 4.68 g/L of D-PLA can be obtained, with a yield of 0.925 g/g PPA.

The strategy of increasing intracellular NADH concentration by co-substrate has been demonstrated (Hou et al., 2019), and glycerol is also commonly used for dehydrogenation to produce lactic acid (Bharath et al., 2020). Therefore, in this study, intracellular NADH concentration was increased by using glycerol as the co-substrate of PPA. The influence of co-substrate on the yield of D-PLA was explored by adjusting the concentration and proportion of PPA and glycerol as shown in Figure 5D. D-PLA titer increased with the increase of PPA concentration, but the yield showed a trend of continuous decline, because the increase of PPA concentration enhanced the toxic effect on *E. coli* CP303. When the concentration of PPA increased to 7–8 g/L, the strong inhibitory effect made the conversion rate of PPA decreased greatly. Therefore, the optimal PPA concentration was 6 g/L. Furthermore, the addition amount of glycerol was optimized. When the glycerol concentration increased from equal molar concentration to 3 times molar concentration (relative to PPA), both the yield of D-PLA and PPA conversion showed a trend of first increasing and then decreasing at the same PPA concentration. This is because the low concentration of glycerol oxidizes itself to dihydroxyacetone through GlyDH and cannot provide sufficient coenzyme NADH, while the high concentration of glycerol has a certain inhibitory effect on the growth of *E. coli* and enzyme activity. Furthermore, without glycerol as the control, the maximum yield of D-PLA was only 4.63 g/L. Therefore, the optimal glycerol dosage was 2 times of the molar concentration of PPA. Under optimal conditions (6 g/L PPA, 6.73 g/L glycerol, 0.2 mM IPTG, pH 7, 25°C) of whole-cell catalytic production for 12 h, the maximum titer 5.87 g/L of D-PLA can be obtained with a yield of 0.967 g/g PPA.

### Scale-Up of D-PLA Production

**FIGURE 6:**
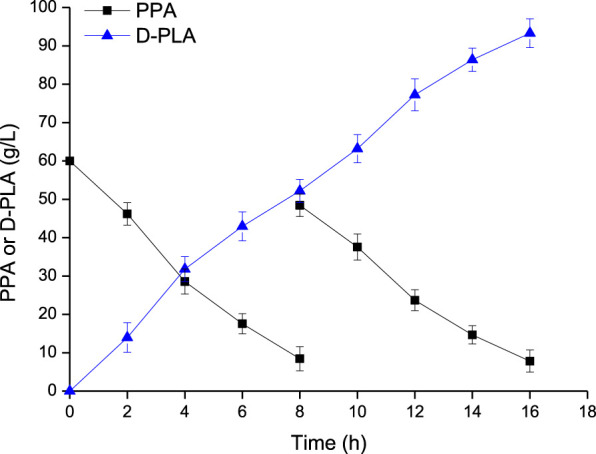
D-PLA production by whole-cell bioconversion using the CP303 strain in a 5 L bioreactor. Data are means ± SD (*n* = 3).

The whole-cell bioconversion was performed in a 5-L reactor using *E. coli* CP303 as biocatalyst as shown in Figure 6. When the fermentation executed for 8 h, and the concentration of substrate PPA and glycerol reduced from 60 g/L to 8.45 g/L and form 66.5 g/L to 37.6 g/L, respectively. Another PPA and glycerol addition into the bioreactor were conducted to reach a level of 48.45 g/L and 54.36 g/L, respectively. The whole-cell catalysis lasted for 16 h, and the final concentration of D-PLA was 93.3 g/L (561.53 mM), with a productivity of 5.83 g/L/h, and yield of 0.922 g/g PPA.

## Conclusion

In this study, we reported for the first time the one-step synthesis of D-PLA using PPA as substrate by co-expression of D-LDH and GlyDH, achieving the regeneration of coenzyme NADH. At the same time, in order to further improve the regeneration efficiency of NADH and the yield of D-PLA, the D-LDH/GlyDH fusion protein strains were constructed with flexible glycine-rich linkers. The results showed that the fusion protein strain CP303 achieved a yield of 0.967 g/g PPA. In our study, cheap glycerol was used as co-substrate and coenzyme NADH was not needed to be added. A high-efficiency and low-cost pathway was constructed to sustainably produce high value-added product D-PLA, which is a technology with industrial potential.

## Data Availability

The original contributions presented in the study are included in the article/Supplementary Material, further inquiries can be directed to the corresponding author.

## Abbreviations

DCW, cell catalyst; D-LDH, d-lactate dehydrogenase; D-PLA, d-phenyllactic acid; FDH, formate dehydrogenase; GDH, glucose dehydrogenase; GlyDH, glycerol dehydrogenase; HPLC-MS, high performance liquid chromatography-mass spectrometry; IPTG, isopropyl-β-d-thiogalactoside; l1, (GGGGS)1; l2, (GGGGS)2; l3, (GGGGS)3; l6, (GGGGS)6; PCR, polymerase chain reaction; PLA, phenyllactic acid; PPA, sodium phenylpyruvate.

## References

[B1] Aalbers F. S. Fraaije M. W. (2017). Coupled Reactions by Coupled Enzymes: Alcohol to Lactone cascade with Alcohol Dehydrogenase-Cyclohexanone Monooxygenase Fusions. Appl. Microbiol. Biotechnol. 101 (20), 7557–7565. 10.1007/s00253-017-8501-4 28916997 PMC5624969

[B2] Aalbers F. S. Fraaije M. W. (2019). Enzyme Fusions in Biocatalysis: Coupling Reactions by Pairing Enzymes. Chembiochem 20 (1), 20–28. 10.1002/cbic.201800394 30178909 PMC6563810

[B3] Abadli M. Dewasme L. Tebbani S. Dumur D. Vande Wouwer A. (2021). An Experimental Assessment of Robust Control and Estimation of Acetate Concentration in *Escherichia coli* BL21(DE3) Fed-Batch Cultures. Biochem. Eng. J. 174, 108103. 10.1016/j.bej.2021.108103

[B4] Anami Y. Yamazaki C. M. Xiong W. Gui X. Zhang N. An Z. (2018). Glutamic Acid-Valine-Citrulline Linkers Ensure Stability and Efficacy of Antibody-Drug Conjugates in Mice. Nat. Commun. 9 (1), 2512. 10.1038/s41467-018-04982-3 29955061 PMC6023893

[B5] Béguin P. (1999). Hybrid Enzymes. Curr. Opin. Biotechnol. 10 (4), 336–340. 10.1016/s0958-1669(99)80061-5 10449312

[B6] Bharath G. Rambabu K. Hai A. Taher H. Banat F. (2020). Development of Au and 1D Hydroxyapatite Nanohybrids Supported on 2D Boron Nitride Sheets as Highly Efficient Catalysts for Dehydrogenating Glycerol to Lactic Acid. ACS Sust. Chem. Eng. 8 (19), 7278–7289. 10.1021/acssuschemeng.9b06997

[B7] Cheng Z. Ran Q. Liu J. Deng X. Qiu H. Jia Z. (2020). Rapid Determination for Benzoic Acid, Sorbic Acid, Phenyllactic Acid, Phenylalanine, and Saccharin Sodium in Vinegar by High-Performance Liquid Chromatography-UV. Food Anal. Methods 13 (8), 1673–1680. 10.1007/s12161-020-01784-6

[B8] Costa J. R. Tonon R. V. Cabral L. Gottschalk L. Pastrana L. Pintado M. E. (2020). Valorization of Agricultural Lignocellulosic Plant Byproducts through Enzymatic and Enzyme-Assisted Extraction of High-Value-Added Compounds: A Review. ACS Sust. Chem. Eng. 8 (35), 13112–13125. 10.1021/acssuschemeng.0c02087

[B9] de Almeida Parizotto L. Krebs Kleingesinds E. Manfrinato Pedrotti da Rosa L. Effer B. Meira Lima G. Herkenhoff M. E. (2021). Increased Glycosylated L-Asparaginase Production through Selection of Pichia pastoris Platform and Oxygen-Methanol Control in Fed-Batches. Biochem. Eng. J. 173, 108083. 10.1016/j.bej.2021.108083

[B10] Dieuleveux V. Lemarinier S. Guéguen M. (1998). Antimicrobial Spectrum and Target Site of D-3-Phenyllactic Acid. Int. J. Food Microbiol. 40 (3), 177–183. 10.1016/s0168-1605(98)00031-2 9620125

[B11] Dueber J. E. Wu G. C. Malmirchegini G. R. Moon T. S. Petzold C. J. Ullal A. V. (2009). Synthetic Protein Scaffolds Provide Modular Control over Metabolic Flux. Nat. Biotechnol. 27 (8), 753–759. 10.1038/nbt.1557 19648908

[B12] Fan L. Wang Y. Tuyishime P. Gao N. Li Q. Zheng P. (2018). Engineering Artificial Fusion Proteins for Enhanced Methanol Bioconversion. Chembiochem 19 (23), 2465–2471. 10.1002/cbic.201800424 30246938

[B13] He R. Finan B. Mayer J. P. DiMarchi R. D. (2019). Peptide Conjugates with Small Molecules Designed to Enhance Efficacy and Safety. Molecules 24 (10), 1855. 10.3390/molecules24101855 PMC6572008 31091786

[B14] Hou Y. Gao B. Cui J. Tan Z. Qiao C. Jia S. (2019). Combination of Multi-Enzyme Expression fine-tuning and Co-substrates Addition Improves Phenyllactic Acid Production with an *Escherichia coli* Whole-Cell Biocatalyst. Bioresour. Tech. 287, 121423. 10.1016/j.biortech.2019.121423 31103936

[B15] Hou Y. Hossain G. S. Li J. Shin H.-D. Du G. Chen J. (2017). Metabolic Engineering of Cofactor Flavin Adenine Dinucleotide (FAD) Synthesis and Regeneration inEscherichia Colifor Production of α-keto Acids. Biotechnol. Bioeng. 114 (9), 1928–1936. 10.1002/bit.26336 28498544

[B16] Iturrate L. Sánchez-Moreno I. Oroz-Guinea I. Pérez-Gil J. García-Junceda E. (2010). Preparation and Characterization of a Bifunctional Aldolase/Kinase Enzyme: A More Efficient Biocatalyst for C-C Bond Formation. Chem. Eur. J. 16 (13), 4018–4030. 10.1002/chem.200903096 20198665

[B17] Jung S. Hwang H. Lee J.-H. (2019). Effect of Lactic Acid Bacteria on Phenyllactic Acid Production in Kimchi. Food Control 106, 106701. 10.1016/j.foodcont.2019.06.027

[B18] Kawaguchi H. Teramura H. Uematsu K. Hara K. Y. Hasunuma T. Hirano K. (2015). Phenyllactic Acid Production by Simultaneous Saccharification and Fermentation of Pretreated Sorghum Bagasse. Bioresour. Tech. 182, 169–178. 10.1016/j.biortech.2015.01.097 25689311

[B19] Lerchner A. Daake M. Jarasch A. Skerra A. (2016). Fusion of an Alcohol Dehydrogenase with an Aminotransferase Using a PAS Linker to Improve Coupled Enzymatic Alcohol-To-Amine Conversion. Protein Eng. Des. Selection 29 (12), 557–562. 10.1093/protein/gzw039 27578886

[B20] Li L. Shin S.-Y. Lee K. W. Han N. S. (2014). Production of Natural Antimicrobial Compound D -phenyllactic Acid Using Leuconostoc Mesenteroides ATCC 8293 Whole Cells Involving Highly Active D -lactate Dehydrogenase. Lett. Appl. Microbiol. 59 (4), 404–411. 10.1111/lam.12293 24888766

[B21] Li X. Jiang B. Pan B. Mu W. Zhang T. (2008). Purification and Partial Characterization of Lactobacillus Species SK007 Lactate Dehydrogenase (LDH) Catalyzing Phenylpyruvic Acid (PPA) Conversion into Phenyllactic Acid (PLA). J. Agric. Food Chem. 56 (7), 2392–2399. 10.1021/jf0731503 18333614

[B22] Lindbladh C. Persson M. Bulow L. Mosbach K. (1992). Characterization of a Recombinant Bifunctional Enzyme, Galactose Dehydrogenase/bacterial Luciferase, Displaying an Improved Bioluminescence in a Three-Enzyme System. Eur. J. Biochem. 204 (1), 241–247. 10.1111/j.1432-1033.1992.tb16630.x 1740135

[B23] Liu J. Huang R. Song Q. Xiong H. Ma J. Xia R. (2021). Combinational Antibacterial Activity of Nisin and 3-Phenyllactic Acid and Their Co-production by Engineered Lactococcus Lactis. Front. Bioeng. Biotechnol. 9, 612105. 10.3389/fbioe.2021.612105 33634085 PMC7901885

[B24] Luo X. Zhang Y. Yin F. Hu G. Jia Q. Yao C. (2020a). Enzymological Characterization of a Novel D-Lactate Dehydrogenase from Lactobacillus Rossiae and its Application in D-Phenyllactic Acid Synthesis. 3 Biotech. 10 (3), 101. 10.1007/s13205-020-2098-5 PMC7005236 32099742

[B25] Luo X. Zhang Y. Yin L. Zheng W. Fu Y. (2020b). Efficient Synthesis of D-Phenyllactic Acid by a Whole-Cell Biocatalyst Co-expressing Glucose Dehydrogenase and a Novel D-Lactate Dehydrogenase from Lactobacillus Rossiae. 3 Biotech. 10 (1), 14. 10.1007/s13205-019-2003-2 PMC6904706 31879578

[B26] Mu W. Yu S. Jiang B. Li X. (2012). Characterization of D-Lactate Dehydrogenase from Pediococcus Acidilactici that Converts Phenylpyruvic Acid into Phenyllactic Acid. Biotechnol. Lett. 34 (5), 907–911. 10.1007/s10529-012-0847-1 22261863

[B27] Patgiri A. Skinner O. S. Miyazaki Y. Schleifer G. Marutani E. Shah H. (2020). An Engineered Enzyme that Targets Circulating Lactate to Alleviate Intracellular NADH:NAD+ Imbalance. Nat. Biotechnol. 38 (3), 309–313. 10.1038/s41587-019-0377-7 31932725 PMC7135927

[B28] Peters C. Rudroff F. Mihovilovic M. D. T. Bornscheuer U. (2017). Fusion Proteins of an Enoate Reductase and a Baeyer-Villiger Monooxygenase Facilitate the Synthesis of Chiral Lactones. Biol. Chem. 398 (1), 31–37. 10.1515/hsz-2016-0150 27289001

[B29] Prachayasittikul V. Ljung S. Isarankura-Na-Ayudhya C. Bülow L. (2006). NAD(H) Recycling Activity of an Engineered Bifunctional Enzyme Galactose Dehydrogenase/lactate Dehydrogenase. Int. J. Biol. Sci. 2 (1), 10–16. 10.7150/ijbs.2.10 16585948 PMC1415851

[B30] Rajanikar R. V. Nataraj B. H. Naithani H. Ali S. A. Panjagari N. R. Behare P. V. (2021). Phenyllactic Acid: A green Compound for Food Biopreservation. Food Control 128, 108184. 10.1016/j.foodcont.2021.108184

[B31] Sorrentino E. Tremonte P. Succi M. Iorizzo M. Pannella G. Lombardi S. J. (2018). Detection of Antilisterial Activity of 3-Phenyllactic Acid Using Listeria Innocua as a Model. Front. Microbiol. 9, 1373. 10.3389/fmicb.2018.01373 29997593 PMC6028618

[B32] Sun H.-L. Chuai J. Wei H. Zhang X. Yu H. (2019). Multi-functional Organic Gelator Derived from Phenyllactic Acid for Phenol Removal and Oil Recovery. J. Hazard. Mater. 366, 46–53. 10.1016/j.jhazmat.2018.11.095 30502572

[B33] Valerio F. Di Biase M. Lattanzio V. M. T. Lavermicocca P. (2016). Improvement of the Antifungal Activity of Lactic Acid Bacteria by Addition to the Growth Medium of Phenylpyruvic Acid, a Precursor of Phenyllactic Acid. Int. J. Food Microbiol. 222, 1–7. 10.1016/j.ijfoodmicro.2016.01.011 26827290

[B34] Wang X. Hou Y. Liu L. Li J. Du G. Chen J. (2018a). A New Approach for Efficient Synthesis of Phenyllactic Acid from L-Phenylalanine: Pathway Design and Cofactor Engineering. J. Food Biochem. 42 (5), e12584. 10.1111/jfbc.12584

[B35] Wang Y. Ren H. Zhao H. (2018b). Expanding the Boundary of Biocatalysis: Design and Optimization of *In Vitro* Tandem Catalytic Reactions for Biochemical Production. Crit. Rev. Biochem. Mol. Biol. 53 (2), 115–129. 10.1080/10409238.2018.1431201 29411648 PMC6112242

[B36] Wu W. Deng G. Liu C. Gong X. Ma G. Yuan Q. (2020). Optimization and Multiomic Basis of Phenyllactic Acid Overproduction by Lactobacillus Plantarum. J. Agric. Food Chem. 68 (6), 1741–1749. 10.1021/acs.jafc.9b07136 31964137

[B37] Wu X. Zhang C. Xing X. H. Yun Z. Zhao L. Wu Q. (2021). Construction and Characterization of Novel Bifunctional Fusion Proteins Composed of Alcohol Dehydrogenase and NADH Oxidase with Efficient Oxidized Cofactor Regeneration. Biotechnol. Appl. Biochem 10.1002/bab.2225 34269481

[B38] Xiong W. Liu B. Shen Y. Jing K. Savage T. R. (2021). Protein Engineering Design from Directed Evolution to De Novo Synthesis. Biochem. Eng. J. 174, 108096. 10.1016/j.bej.2021.108096

[B39] Xu G.-C. Zhang L.-L. Ni Y. (2016). Enzymatic Preparation of D-Phenyllactic Acid at High Space-Time Yield with a Novel Phenylpyruvate Reductase Identified from Lactobacillus Sp. CGMCC 9967. J. Biotechnol. 222, 29–37. 10.1016/j.jbiotec.2015.12.011 26712480

[B40] Xu J. J. Fu L. J. Si K. L. Yue T. L. Guo C. F. (2020). 3‐phenyllactic Acid Production by Free‐whole‐cells of Lactobacillus Crustorum in Batch and Continuous Fermentation Systems. J. Appl. Microbiol. 129 (2), 335–344. 10.1111/jam.14599 32009287

[B41] Xu J. Xu X. Wang Q. Fan X. (2015). Chiral Separation of Phenyllactic Acid by Helical Structure from spring Dextrin. J. Incl Phenom Macrocycl Chem. 82 (3-4), 515–521. 10.1007/s10847-015-0487-x

[B42] Yang H. Liu L. Shin H.-d. Chen R. R. Li J. Du G. (2013). Comparative Analysis of Heterologous Expression, Biochemical Characterization Optimal Production of an Alkaline α-amylase from alkaliphilicAlkalimonas amylolyticainEscherichia coliandPichia Pastoris. Biotechnol. Prog. 29 (1), 39–47. 10.1002/btpr.1657 23125186

[B43] Yu K. Liu C. Kim B.-G. Lee D.-Y. (2015a). Synthetic Fusion Protein Design and Applications. Biotechnol. Adv. 33 (1), 155–164. 10.1016/j.biotechadv.2014.11.005 25450191

[B44] Yu S. Zhou C. Zhang T. Jiang B. Mu W. (2015b). 3-Phenyllactic Acid Production in Milk by SK25 during Laboratory Fermentation Process. J. Dairy Sci. 98 (2), 813–817. 10.3168/jds.2014-8645 25434344

[B45] Yu S. Zhu L. Zhou C. An T. Jiang B. Mu W. (2014). Enzymatic Production of D-3-Phenyllactic Acid by Pediococcus Pentosaceus D-Lactate Dehydrogenase with NADH Regeneration by Ogataea Parapolymorpha Formate Dehydrogenase. Biotechnol. Lett. 36 (3), 627–631. 10.1007/s10529-013-1404-2 24249102

[B46] Zhang X. X. Abd Elazim A. M. Ruiz G. Yu R. C. (2014). Fracture Behaviour of Steel Fibre-Reinforced concrete at a Wide Range of Loading Rates. Int. J. Impact Eng. 71, 89–96. 10.1016/j.ijimpeng.2014.04.009

[B47] Zhang Y. Wang Y. Wang S. Fang B. (2017). Engineering Bi-functional Enzyme Complex of Formate Dehydrogenase and Leucine Dehydrogenase by Peptide Linker Mediated Fusion for Accelerating Cofactor Regeneration. Eng. Life Sci. 17 (9), 989–996. 10.1002/elsc.201600232 32624849 PMC6999412

[B48] Zhao W. Ding H. Lv C. Hu S. Huang J. Zheng X. (2018). Two-step Biocatalytic Reaction Using Recombinant *Escherichia coli* Cells for Efficient Production of Phenyllactic Acid from L-Phenylalanine. Process Biochem. 64, 31–37. 10.1016/j.procbio.2017.09.019

[B49] Zheng Z. Xia M. Fang X. Jiang T. Ouyang J. (2018). Enhanced Biosynthesis of Chiral Phenyllactic Acid from L-Phenylalanine through a New Whole-Cell Biocatalyst. Bioproc. Biosyst Eng 41 (8), 1205–1212. 10.1007/s00449-018-1949-5 29931478

[B50] Zhu Y. Wang Y. Xu J. Chen J. Wang L. Qi B. (2017). Enantioselective Biosynthesis of L-Phenyllactic Acid by Whole Cells of Recombinant *Escherichia coli* . Molecules 22 (11), 1966. 10.3390/molecules22111966 PMC6150373 29140277

