# Supplementary material for: Using Unnatural Protein Fusions to Engineer a Coenzyme Self-Sufficiency System for D-Phenyllactic Acid Biosynthesis in Escherichia coli
Source: Front Bioeng Biotechnol. 2021 Dec 17;9:795885. doi: 10.3389/fbioe.2021.795885 (PMC8718758; doi:10.3389/fbioe.2021.795885)
Supplement: Supplementary file 1 [file DataSheet1.docx]

**SUPPLEMENTARY MATERIAL**

**Using unnatural protein fusions to engineer a coenzyme self-sufficiency system for D-phenyllactic acid biosynthesis in *Escherichia coli***

**Zhao Qin^1^, Dan Wang^1*^, Ruoshi Luo^1^, Tinglan Li^1^, Xiaochao Xiong^2^, Peng Chen^1^**

**^1^School of Chemistry and Chemical Engineering, Chongqing University, Chongqing, 400044, P.R. China**

**^2^Department of Biological Systems Engineering, Washington State University, Pullman, WA 99164-6120, USA**

***Corresponding author**

**Dr. Dan Wang (dwang@cqu.edu.cn);**

**School of Chemistry and Chemical Engineering**

**Chongqing University**

**Chongqing, 400044, P.R. China**

**Tel: (86) 18523118282**

|  |  |
| --- | --- |
|  | |

**Figure** **S1**. HPLC confirmation of D-PLA biosynthesis by recombinant *E.coli* BL21(DE3). A: D-PLA standard sample. B: PPA standard sample. C: D-PLA and PLA fermentation broth

|  |  |
| --- | --- |

**Figure** **S2.** MS analysis of D-PLA produced by recombinant *E.coli* BL21(DE3). A: Mass spectrum of D-PLA standard sample, B: Mass spectrum of D-PLA form fermentation broth

**Supplementary** **Table 1** Strains and plasmids used in this study

| Plasmids or Strains | Relevant characteristics | Source |
| --- | --- | --- |
| pET28a | Kan,T7 promoter | Laboratory storage |
| pET28a-*LfD-LDH* | pET28a carries a *D-LDH* gene from *Lactobacillus fermentum* | This study |
| pET28a-*LsD-LDH* | pET28a carries a *D-LDH* gene from *Lactobacillus sp*.SK007 | This study |
| pET28a-*BmGlyDH* | pET28a carries a *GlyDH* gene from *Bacillus megaterium* | This study |
| pET28a-*EsGlyDH* | pET28a carries a *GlyDH* gene from *Exiguobacterium sibiricum* | This study |
| *E. coli* BL21(DE3) | Wild type | Laboratory storage |
| CP100 | *E. coli* BL21(DE3) harboring pET28a | This study |
| CP101 | *E. coli* BL21(DE3) harboring pET28a-*LfD-LDH* | This study |
| CP102 | *E. coli* BL21(DE3) harboring pET28a-*LsD-LDH* | This study |
| CP103 | *E. coli* BL21(DE3) harboring pET28a-*BmGlyDH* | This study |
| CP104 | *E. coli* BL21(DE3) harboring pET28a-*EsGlyDH* | This study |
| CP201 | *E. coli* BL21(DE3) harboring pET28a-*LfD-LDH-BmGlyDH* | This study |
| CP202 | *E. coli* BL21(DE3) harboring pET28a-*LfD-LDH-EsGlyDH* | This study |
| CP203 | *E. coli* BL21(DE3) harboring pET28a-*LsD-LDH-BmGlyDH* | This study |
| CP204 | *E. coli* BL21(DE3) harboring pET28a-*LsD-LDH-EsGlyDH* | This study |
| CP301 | *E. coli* BL21(DE3) harboring pET28a-*LfD-LDH-l_1_-BmGlyDH* | This study |
| CP302 | *E. coli* BL21(DE3) harboring pET28a-*LfD-LDH-l_2_-BmGlyDH* | This study |
| CP303 | *E. coli* BL21(DE3) harboring pET28a-*LfD-LDH-l_3_-BmGlyDH* | This study |
| CP304 | *E. coli* BL21(DE3) harboring pET28a-*LfD-LDH-l_6_-BmGlyDH* | This study |

**Supplementary** **Table 2** Linkers used in this study

| Linkers | Linkers sequence |
| --- | --- |
| l_1_: (GGGGS)_1_ | GGCGGTGGTGGCTCC |
| l_2_: (GGGGS)_2_ | GGCGGTGGTGGCTCCGGTGGTGGTGGCTCC |
| l_3_: (GGGGS)_3_ | GGCGGTGGTGGCTCCGGTGGTGGTGGCTCCGGCGGCGGCGGTTCT |
| l_6_: (GGGGS)_6_ | GGCGGTGGTGGCTCCGGTGGTGGTGGCTCCGGCGGCGGCGGTTCTGGTGGCGGCGGTTCCGGCGGTGGCGGTTCCGGTGGTGGCGGCTCT |

**Supplementary** **Table 3** Primers used in this study

| Primers | Nucleotide sequences（5’-3’） | Linker length |
| --- | --- | --- |
| *D-LDH-*F | CGGGATCCATGGCAAAAATTTACGCATACGG |  |
| *D-LDH-*R | TGATTGAATAATGCGGTCCATTTAACCAACCTTAACTGGGGTTTC |  |
| *GlyDH*0-F | GAAACCCCAGTTAAGGTTGGTATGGACCGCATTATTCAATCACCG |  |
| *GlyDH*1-F | GAAACCCCAGTTAAGGTTGGTGGCGGTGGTGGCTCCATGGACCGCATTATTCAA | (GGGGS)_1_ |
| *GlyDH*2-F | GAAACCCCAGTTAAGGTTGGTGGCGGTGGTGGCTCCGGTGGTGGTGGCTCCATGGACCGCATTATTCAATCACCG | (GGGGS)_2_ |
| *GlyDH*3-F | GAAACCCCAGTTAAGGTTGGTGGCGGTGGTGGCTCCGGTGGTGGTGGCTCCGGCGGCGGCGGTTCTATGGACCGCATTATTCAATCACCG | (GGGGS)_3_ |
| *GlyDH*4-F | GAAACCCCAGTTAAGGTTGGTGGCGGTGGTGGCTCCGGTGGTGGTGGCTCCGGCGGCGGCGGTTCTGGTGGCGGCGGTTCCGGCGGTGGCGGTTCCGGTGGTGGCGGCTCTATGGACCGCATTATTCAATCACCG | (GGGGS)_6_ |
| *GlyDH*0-R/1-R/2-R/3-R/4-R | CCGCCTCGAGTTATTCCCACTCTTGCAG |  |
